# Long-term efficacy and safety of peptide receptor radionuclide therapy in Japanese patients with unresectable neuroendocrine tumor: extension of the Japanese phase I and phase I/II study

**DOI:** 10.1007/s12149-026-02169-1

**Published:** 2026-02-12

**Authors:** Noritoshi Kobayashi, Hiroaki Ono, Naoki Okubo, Keiichi Akahoshi, Atsushi Kudo, Daisuke Ban, Yasushi Ichikawa

**Affiliations:** 1 Department of Oncology, Yokohama City University Graduate School of Medicine, 3-9 Fukuura Kanazawa-ku, Yokohama, 236-0004 Japan; 2 Department of Hepatobiliary and Pancreatic Surgery, Institute of Science Tokyo, Tokyo, Japan

**Keywords:** Peptide receptor radionuclide therapy, Neuroendocrine tumors, Japan

## Abstract

**Objective:**

Peptide receptor radionuclide therapy (PRRT) with radiolabeled somatostatin analogs is an innovative treatment for advanced well-differentiated neuroendocrine tumors (NETs). In Japan, PRRT with Lutetium-177 oxodotreotide (^177^Lu- oxodotreotide) was recently approved following Phase I and Phase I/II clinical trials, which primarily assessed safety and response rates. However, its long-term efficacy and safety remain unclear. We conducted a record-based analysis of these trials to evaluate progression-free survival (PFS), overall survival (OS), and long-term safety.

**Methods:**

We recruited Japanese patients from the Phase I and Phase I/II clinical trials. Patients received four infusions of 7.4 GBq ^177^Lu-oxodotreotide every 8 weeks. We reviewed the database records to assess OS, disease progression, and adverse events.

**Results:**

Twenty-one patients (median age, 57 years) were recruited. The primary tumor sites included the pancreas (n=11), small intestine (n=5), rectum (n=2), lungs (n=2), and stomach (n=1). Twenty (95.2%) patients had liver metastases, whereas 19 (90.5%) had grade 2 tumors. The median PFS period was 36.6 months; median OS period was not　reached. Two (9.5%) patients achieved a complete response, 12 (57.1%) achieved a partial response, six (28.6%) had stable disease, and one (4.8%) had progressive disease. One patient had chronic myeloid leukemia 15 months post-PRRT.

**Conclusions:**

PRRT demonstrated sustained efficacy and safety in Japanese patients with NET.

**Supplementary Information:**

The online version contains supplementary material available at 10.1007/s12149-026-02169-1.

## Introduction

Neuroendocrine neoplasms (NENs) are malignant tumors with neuroendocrine differentiation that can develop in various organs throughout the body. In recent pathological classifications, these tumors are defined as those expressing biomarkers related to secretion from normal neuroendocrine cells or neurons associated with large dense core vesicles and small synaptic-like vesicles (SSVs) [1]. Within NENs, well-differentiated neuroendocrine tumors (NETs) exhibit epithelial differentiation and share many features of normal neuroendocrine cells, and they occur predominantly in the gastrointestinal tract and respiratory system [2]. Clinically, these tumors are rare and often progress slowly; however, once metastatic they can become fatal because curative treatment options are limited [3]. They can also present with hormonal symptoms that substantially impair patients’ quality of life, making them clinically distinct among solid malignancies.

Notably, the primary organ in which NETs occur differs among races. In Japanese patients, rectal NETs are more common than small intestinal NETs, whereas in Western cohorts small intestinal (midgut) NETs are relatively more prevalent [4]. This finding highlights the importance of generating Japan-specific clinical data, because direct extrapolation from Western cohorts may be misleading. Surgical resection is the only curative treatment; nonetheless, the prognosis of patients with metastatic or recurrent lesions is poor. Treatments for metastatic or recurrent cases have rapidly advanced in Western countries. The main pharmacological treatments include long-acting somatostatin analogs (octreotide and lanreotide) and the molecularly targeted drugs everolimus and sunitinib, which have been developed over the past decade and are now widely used in Japan [5–7]. Peptide receptor radionuclide therapy (PRRT) targets somatostatin receptors that are highly expressed on NET cell membranes and offers a favorable antitumor efficacy–toxicity balance compared with conventional systemic therapies. PRRT has been clinically used in Europe for approximately 30 years [8, 9]. In 2017, treatment with Lutetium-177 oxodotreotide (^177^Lu-oxodotreotide) plus octreotide long-acting release (LAR) was shown in the NETTER-1 phase III trial to improve progression-free survival in patients with advanced midgut NETs, leading to its regulatory approval as a treatment for unresectable or metastatic, progressive, well-differentiated gastroenteropancreatic (GEP)-NETs in Europe and the US [10].

In Japan, patients with NETs historically traveled to Europe to receive PRRT; however, a corporate bridging program with phase I and phase I/II trials of ^77^Lu- oxodotreotide was initiated by FUJIFILM RI Pharma Co., Ltd., and based on these data PRRT with ^177^Lu- oxodotreotide received regulatory approval in June 2021 [11–13]. The primary endpoint of the Phase I study was safety, comprising patients with advanced pancreatic and gastrointestinal NETs [12]. The safety of radioactivity was evaluated by accumulating data, such as the blood kinetics of radioactivity, urinary excretion rate, radiation dose to each major organ, hematologic toxicity to patients, and non-hematologic toxicity. In the Phase I/II study, the primary endpoint was efficacy in response rate (RR), as well as safety in patients with somatostatin receptor-positive advanced midgut carcinoids and other NETs [13]. The results confirmed a high RR of 66.7% with a favorable safety profile. Based on these results, FUJIFILM RI Pharma Co., Ltd. applied to the Pharmaceuticals and Medical Devices Agency for approval, which led to domestic approval of the target drug, and the clinical trial was completed in March 2021.

Initially, PRRT was mainly used as a second- or later-line treatment for unresectable pancreatic and gastrointestinal NETs [14]. However, recent NETTER-2 phase III data have shown improved response rates and significantly prolonged progression-free survival when ^177^Lu- oxodotreotide plus octreotide LAR is used as first-line therapy in patients with advanced, somatostatin receptor (SSTR)-positive, grade 2–3 GEP-NETs [15]. Consequently, PRRT is now the first-line treatment for unresectable NETs in Japanese guidelines [16]. However, these results were based on patients from Western countries. The long-term efficacy and safety of this therapy in Japanese patients have not been confirmed.

Therefore, in this study, we aimed to clarify the long-term role of PRRT in Japanese patients by evaluating the long-term treatment effects and safety in patients who participated in domestic clinical trials.

## Materials and methods

This study was designed as a retrospective observational cohort study of Japanese patients who had participated in phase I and phase I/II clinical trials of ^177^Lu- oxodotreotide; data were collected from their medical records up to August 2023. This retorospective study was reviewed and approved by the Institutional Review Boards at Yokohama City University Hospital and Tokyo Medical Dental University Hospital (F230900001). This study was conducted in accordance with the rules and principles of the 1964 Declaration of Helsinki and its subsequent amendments. Owing to the retrospective nature of the study, we applied the opt-out method for obtaining consent to participate by stating on the website approved by the Institutional Review Board.

We recruited Japanese patients who participated in a Phase I clinical trial of ^177^Lu- oxodotreotide for advanced pancreatic, gastrointestinal, or pulmonary NETs that were somatostatin receptor-positive (protocol number P-1515-11), as well as a Phase I/II clinical trial of ^177^Lu- oxodotreotide for progressive midgut carcinoids and other somatostatin receptor-positive NETs (protocol number P-1515-12).

The primary endpoint was progression-free survival (PFS). Secondary endpoints included overall survival (OS); incidence of therapy-related myeloid neoplasms (including myelodysplastic syndrome, acute leukemia, and other hematologic malignancies); longitudinal changes in bone marrow function (leukocytes, neutrophils, lymphocytes, hemoglobin, and platelets); longitudinal changes in renal function (serum creatinine levels, creatinine clearance using the Cockcroft–Gault formula, and estimated glomerular filtration rate), and other serious events and diseases.

The main inclusion criteria used in both clinical trials were as follows: confirmed SSTR-positive, unresectable, progressive pancreatic, gastrointestinal, or pulmonary NET with a Ki-67 index ≤20% (WHO grade 1–2). Standard therapies were either ineffective, or no other adequate treatment options were available. Tumor progression, as defined by the RECIST criteria version 1.1, was confirmed by comparing the most recent computed tomography/magnetic resonance imaging (CT/MRI) scan obtained within 12 months before enrollment to a scan from any time in the past. Efficacy outcomes were assessed based on the best overall response, defined as the maximum tumor response achieved at any time after the initiation of PRRT according to RECIST 1.1. The median duration from the first PRRT administration to the first imaging confirmation of objective response (CR or PR) was defined as median response time, and the median time from the first documented objective response (CR or PR) until objective tumor progression or death was defined as the median duration of response. The presence of SSTRs on all target lesions documented using CT/MRI scans was evaluated based on a positive somatostatin receptor scintigraphy (SRS) performed within 4 months before the study enrollment. The tumor uptake observed in each target lesion must be equal to or higher than the normal liver uptake observed on planar imaging using the modified Krenning scale.

The patients had adequate organ functions if the following criteria were met: hemoglobin (Hb) concentration ≥8.0 g/dL, white blood cell (WBC) count ≥2,000/μL, platelet count ≥7.5 × 10^4^/μL, serum creatinine levels ≤1.7 mg/dL, creatinine clearance ≥50 mL/min, total bilirubin levels ≤3 × ULN, serum albumin levels >3.0 g/dL, and an Eastern Cooperative Oncology Group (ECOG) Performance Status 0 or 1. Japanese patients aged ≥20 years at the time of consent were eligible for the study.

Cases that were not registered in these clinical trials were excluded. Additionally, the exclusion criteria were the same as those used in each clinical trial. The main exclusion criteria were poorly differentiated NET and neuroendocrine, small-cell, and large-cell neuroendocrine carcinomas. The exclusion criteria also included pre-existing severe hematological toxicities, renal toxicities, liver toxicities, uncontrollable diabetes mellitus, and severe concomitant illnesses, including severe psychiatric disorders. We also excluded patients receiving systemic drug therapy, including everolimus, sunitinib malate, streptozocin, or other antineoplastic drugs, within 8 weeks before enrollment, and those receiving operative therapy, radiofrequency ablation, chemoembolization, or radioembolization therapy within 12 weeks before enrollment. Patients who had undergone PRRT at least once and those with ≥25% of the bone marrow and external-beam radiation therapy cases were excluded.

^177^Lu- oxodotreotide was administered at a dose of 7.4 GBq using intravenous infusion combined with 1,000 mL of amino acid solution infusion. Patients received up to four doses every 8 weeks. The dosing interval was extended to 16 weeks to allow recovery from toxicity.

Additionally, sustained-release octreotide 30 mg was intramuscularly administered to patients in the gluteal region on the day following each ^177^Lu- oxodotreotide administration in the Phase I study and midgut NET cases in the Phase I/II study (Protocol No.: P-1515-12).

### Statistical analysis

Statistical analysis was performed using the SPSS Software Package (SPSS Version 25; IBM Corp., Armonk, NY, USA). The OS and PFS were evaluated using the Kaplan–Meier curve method, and correlation analysis was performed using the log-rank test. Assessment of the effect of multiple parameters (primary lesion, Ki67 index, and maximum liver tumor size) on PFS and OS was performed using the Kaplan–Meier curve method. The median follow-up time and OS were calculated from the date of the first PRRT cycle, whereas PFS was calculated from the time of the first PRRT until progression and the start of salvage therapy. Differences between the groups were considered significant at p < 0.05. Data are presented as either mean or median values with standard deviations.

## Results

### Patient’s characteristics

Between August 2017 and October 2020, 21 Japanese patients, comprising nine men and 12 women, were enrolled in the study (median age, 57 years; range, 25–73 years). All patients had an ECOG performance status score of 0. The primary tumor lesions observed were in the pancreas (n=11), small intestine (n=5), rectum (n=2), lungs (n=2), and stomach (n=1). Following the pathological grade of the WHO classification 2017/2019, two cases were grade 1, and 19 cases were grade 2, with no grade 3 cases. The mean Ki67 index was 7.6% (range, 1–18.6%). Twenty patients (95.2%) had multiple liver metastatic lesions, and eleven (52.4%) underwent surgical resection for primary lesions.

The median duration from diagnosis to initial PRRT was 38.8 months (range 10.0–314.7 months), and all patients had previously received some systemic treatment. Nineteen patients (90.5%) received a somatostatin analog, seven (33.3%) received everolimus, and four (19.0%) underwent chemotherapy before PRRT. Krenning’s scale score of the most recent SRS was grade 4 (very intense uptake, > kidneys and spleen) in sixteen cases, grade 3 (intense uptake, > liver) in four cases, and grade 2 (moderate uptake, = liver) in one case. Patient characteristics are shown in Table 1.

**Table 1:** Patients’ characteristics

Sex; Male/Female, n (%)	9 (42.9)/12 (57.1)
Age (year), median (range)	57 (25–73)
Primary tumor site, n (%)	
Pancreas	11 (52.4)
Small intestine	5 (23.8)
Rectum	2 (9.5)
Lung	2 (9.5)
Stomach	1 (4.8)
Performance Status, n	
0	21
1	0
Pathological classification (WHO 2019), n (%)	
Grade 1	2 (9.5)
Grade 2	19 (90.5)
Grade 3	0 (0)
Ki67 Labelling index (%) mean (range)	7.6 (1–18.6)
Non-function/function, n (%) Gastrinoma Carcinoid syndrome	18 (85.7)/3 (14.3) 2 (9.5) 1 (4.8)
Metastatic lesions, n (%)	
Liver	20 (95.2)
Bone	3 (14.3)
Lymph node	4 (19.0)
Liver tumor burden, n (%)	
≤25%	18 (90.0)
25%–50%	2 (10)
≥50%	0 (0)
Maximum tumor diameter in liver (mm)	
Median (range)	32 (10–62)
SRS, Krenning scale, n (%)	
Score 2	1 (4.8)
Score 3	4 (19.0)
Score 4	16 (76.2)
Period from diagnosis to PRRT (month) median (range)	38.8 (10.0–314.7)
Previous treatment, n (%)	
Surgical resection	11 (52.4)
Somatostatin analogue	19 (90.5)
Everolimus	7 (33.3)
Sunitinib	7 (33.3)
Chemotherapy	4 (19.0)
TACE	4 (19.0)

SRS: Somatostatin receptor scintigraphy, TACE: Trans arterial chemo embolization

### Treatment

All patients received ^177^Lu-oxodotreotide at 7.4 GBq; however, one patient experienced dose-limited toxicity after the third administration (Table 2). This patient received the fourth ^177^Lu-oxodotreotide at a half-dose of 3.7 GBq. Nineteen patients (90.4%) received PRRT four times as initially planned. Nevertheless, two patients could not complete all four cycles of treatment; one patient received PRRT twice because of tumor progression, and one patient received it three times owing to the inability to order drugs because of the COVID-19 pandemic. The median total dose of ^177^Lu-oxodotreotide was 29.6 GBq (range 7.4–29.6), whereas the dose of administration per single injection was 7.4 GBq (range 3.7–7.4).

**Table 2:** Treatment outcome

Number of 177Lu-Oxodotreotide cycles, n (%)	
1 cycle	0 (0%)
2 cycles	1 (4.8%)
3 cycles	1 (4.8%)
4 cycles	19 (90.4%)
Dose of ^177^Lu-Oxodotreotide median	
Cumulative dose (GBq)	29.6 (7.4–29.6)
Dose per administration (GBq/cycle)	7.4 (3.7–7.4)

GBq: Giga Becquerel

### Efficacy

All patients were assessed for imaging responses. The overall RR was 66.7% in all patients (complete response (CR): n=2, 9.5%; partial response (PR): n=12, 57.1%) (Table 3). Another 28.6% of the patients (n=6) showed stable disease (SD), and the disease control rate was 95.2%. The median response time was 9.7 months (range: 5.6–29.1 months), and the median duration of response was 25.1 months (range: 1.5–65.4 months).

**Table 3:** Efficacy of PRRT in all cases by RECIST criteria version 1.1

Best overall response, n (%)	
CR	2 (9.5)
PR	12 (57.1)
SD	6 (28.6)
PD	1 (4.8)
Unknown	0 (0)
ORR, n (%) DCR, n (%)	14 (66.7) 20 (95.2)

CR: Complete response, PR: Partial response, SD: Stable disease, PD: Progressive disease, ORR: Overall response rate, DCR: Disease control rate

In patients with pancreatic NETs, the RR was 72.7% (CR: n=1; PR: n=7; SD: n=2; Progressive Disease (PD): n=1). In patients with gastrointestinal NETs, the RR was 62.5% (CR: n=1; PR: n=4; SD: n=3; PD: n=0). In patients with pulmonary NETs, the RR was 50% (CR: n=0; PR: n=1; SD: n=1; PD: n=0). In patients with rectal NETs, the RR was 100% (CR: n=0; PR: n=2; SD: n=0; PD: n=0). In patients with gastric NETs, the RR was 100.0% (CR: n=0; PR: n=1; SD: n=0; PD: n=0). In patients with small intestinal NETs, the RR was 40.0% (CR: n=1; PR: n=1; SD: n=3; PD: n=0) (Supplementary Table 1).

The median observation duration from the start of treatment was 57.3 months (range: 8.3–76.8 months). Twelve cases progressed, while seven cases did not, during the data cut-off period. Two cases required alternative treatments instead of being controlled by PRRT. One patient underwent pancreatoduodenectomy with partial liver resection as a conversion surgery approximately 2 years after the fourth PRRT. The efficacy of PRRT was evaluated as a partial response in this case. Another patient received everolimus as an additional treatment and was evaluated as having stable disease 4 years after the final PRRT.

**Fig. 1:**
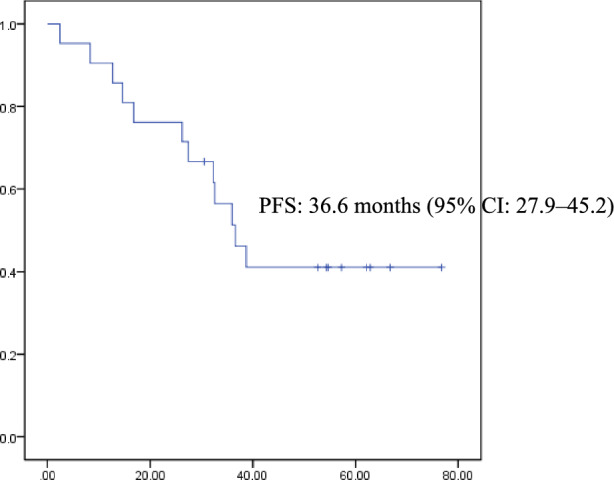
Kaplan–Meier curve of progression free survival with PRRT for all cases. The median time for progression free survival was 36.6 months (95% confidence interval (CI): 27.9–45.2) in all cases

The median time for PFS was 36.6 months (95% confidence interval (CI): 27.9–45.2) in all cases (Fig. 1).

Median PFS period was not reached in patients with pancreatic NETs during the follow-up period. Nonetheless, it was 35.9 months (95% CI 30.4–41.4 months) in patients with gastrointestinal NETs. However, there was no significant difference between the two groups (*P*=0.704) (Online Resource 1). Median PFS period was 36.6 months (95% CI 23.1–50.0 months) in patients with a high Ki67 index, whereas in those with a low Ki67 index, it was 35.9 months. Similarly, no significant difference was found between the two groups (*P*=0.579) (Online Resource 2). Median PFS period was 26.2 months (95% CI: 2.2–50.2 months) in patients with maximum tumor size >3 cm (n=10). Meanwhile, patients with a maximum tumor size of <3 cm (n=11) did not achieve it. There was also no significant difference between the two groups (*P*=0.213) (Online Resource 3). Furthermore, Median PFS period did not significantly differ between the groups, whether PRRT followed surgical resection or not (not reached vs. 36.6 months; 95% CI, 24.9–48.2 months; *P*=0.488). It also did not significantly differ between patients who received SSA maintenance treatment and those who did not. (36.6 months; 95% CI, 28.1–45.1 months vs 32.6 months; *P*=0.712). Moreover, median PFS period was not significantly different, whether PRRT followed targeted molecular therapy or not (not reached vs. 27.4 months; 95% CI 10.4–44.5 months; *P*=0.114). It also did not significantly differ whether PRRT followed chemotherapy or not (not reached vs. 35.9 months; 95% CI 30.4–41.5 months; *P*=0.533).

**Fig. 2:**
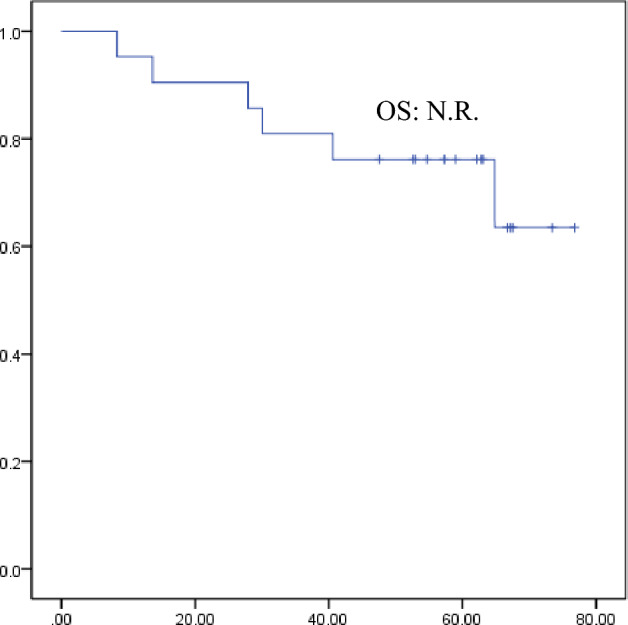
Kaplan-Meier curve of overall survival with PRRT for all cases. Median overall survival was not reached in all cases

The median OS period was not reached in the overall population or in any subgroup during the follow-up period (Fig. 2). Patients with pancreatic and those with gastrointestinal NETs also did not achieve OS, and the two groups did not significantly differ (*P*=0.954) (Online Resource 4). Similarly, Median OS period was not reached in patients with high Ki67 index and low Ki67 index, with no significant difference observed between the two groups (*P*=0.982). A similar trend was observed for patients with maximum tumor sizes of >3 cm and <3 cm (n=11). There was also no significant difference between the two groups (*P*=0.133). The same trend was observed, whether PRRT followed surgical resection or not (*P*=0.748), in patients with SSA maintenance treatment or without (*P*=0.697), whether PRRT followed molecular targeted therapy or not (not reached vs.64.9 months; 95% CI, 20.5–109.2 months; *P*=0.115), and for PRRT, whether followed by cytotoxic chemotherapy or not (*P*=0.553).

### Adverse events

Adverse events were evaluated using the Common Terminology Criteria for Adverse Events version 4.0. Table 4 shows the long-term hematological and renal toxicities. In all grades, 17 (81.0%) anemia, nine (42.9%) lymphocytopenia, six (28.6%) leukocytopenia, and four (19.0%) thrombocytopenia events were observed as hematological toxicities (Online Resource 5 and 6). The most frequently observed severe toxicities were lymphocytopenia (n=3, 14.3%) and anemia (n=2, 9.5%). In all grades, an increase in serum creatinine was observed in five (23.8%) cases, and an e-GFR increase was observed in 15 (71.4%) cases (Online Resource 7 and 8). However, severe renal toxicities were not observed, and there were no cases of treatment-related death.

**Table 4:** Adverse event of PRRT by CTCAE version 4.0

	Grade 1 n (%)	Grade 2 n (%)	Grade 3 n (%)	Grade 4 n (%)
Neutropenia	2 (9.5)	4 (19.0)	0 (0)	0 (0)
Leukocytopenia	0 (0)	2 (9.5)	0 (0)	0 (0)
Anemia	9 (42.9)	6 (28.6)	2 (9.5)	0 (0)
Thrombocytopenia	3 (14.3)	1 (4.8)	0 (0)	0 (0)
Lymphopenia	0 (0)	6 (28.6)	3 (14.3)	0 (0)
Creatinine increased	4 (19.0)	1 (4.8)	0 (0)	0 (0)
eGFR decreased	6 (28.6)	9 (42.9)	0 (0)	0 (0)

eGFR: estimated Glomerular Filtration Rate

Chronic myeloid leukemia (CML) occurred as an unpredictable, severe disease. This case was observed in a 40-year-old male with pancreatic NET Grade 2. Nevertheless, the complaint was not severe, and the patient’s white blood cell count gradually increased 1 year after the fourth PRRT. A bone marrow biopsy was conducted, and the BCR-ABL fusion gene was detected and diagnosed as CML 21 months after the final PRRT. The patient received Dasatinib (Tyrosine-Kinase Inhibitor against BCR-ABL). Subsequently, the condition was controlled, and the white blood cells were within the normal range. The tumors in the liver of the patient gradually shrank, and a partial response was achieved. Disease control continued until the data cut-off period.

## Discussion

In this clinical trial, the therapeutic and side effects of PRRT were observed over a long period of time using data from domestic clinical trials involving Japanese patients with NETs of the pancreas, gastrointestinal tract, and lungs that progressed after receiving standard drug treatment. Over 50% of patients underwent surgical resection. In addition, those who had received some type of systemic treatment were eligible, and despite the patients having a long clinical course of 38.8 months from diagnosis, the RR was high at 66.7%. Furthermore, the long-term disease was controlled, and the median PFS period exceeded 3 years. The RR and PFS were better than those observed in several previous clinical trials [10, 15, 17, 18]. RR was reported to be extremely high in the original study; however, it is commendable that the present study revealed that the anti-tumor effect continued for a long time and that subsequent treatment options were increased, which was confirmed because some patients developed CR during this observation period, and some cases required conversion surgery.

One limitation of this study was that the pancreas, gastrointestinal tract, and lungs all had heterogeneous backgrounds. Moreover, the gastrointestinal tract has a mixture of primary midgut tumors (with a good prognosis) and rectal primary tumors (with a poor prognosis). In addition, for the midgut tumors in the Phase I and Phase I/II studies, the use of SSA was mandatory after treatment, whereas it was not mandatory in other cases. Nonetheless, in many cases, follow-up is performed without treatment. The second limitation of this study was that maintenance treatment was not even because there were only a few studies with a high level of evidence at the time this study was planned. In the NETTER1 trial on midgut tumors, SSAs were required after treatment. In contrast, in the Erasums trial, a Phase I and II study involving gastroenteropancreatic NETs, including midgut NETs, did not require the use of SSAs [10, 17]. Nevertheless, considering the recent Phase III study, NETTER2, in which maintenance treatment with SSA, implemented as the standard of care, showed positive results, it is expected that SSA will be established as maintenance therapy following PRRT [15]. The third limitation of this study was that the follow-up method and period after the end of the clinical trial were not constant, and the evaluation was not uniform.

However, the side effects during the long-term follow-up period were also relatively good. In the long-term evaluation of hematological toxicity, 9.5% of the patients had severe anemia. Grade 3 and 4 anemia was not observed during short-term follow-up; however, it may appear long-term, so caution should be exercised [10, 12, 13]. These data show that an association with extended red blood cell lifespan should also be considered [19]. In contrast, 14.3% of patients had grade 3 and 4 lymphopenia, which was lower than the short-term data of 38.1%, 33.3%, and 46.7% that were previously reported [10, 12, 13], suggesting that lymphopenia may not be prolonged; hence, it may not be necessary to postpone or easily discontinue treatment. Moreover, in the original clinical trials, it was not recommended to postpone or discontinue treatment because of lymphopenia. We observed one case of CML, and previous reports showed that CML is a rare event after PRRT [20–22]. It may have been difficult to prove a causal relationship between PRRT and CML in this case. Bergsma et al. reported that 11 (4%) of the 274 patients had persistent hematologic dysfunction myelodysplastic syndrome (MDS), acute myeloid leukemia (AML), myeloproliferative neoplasm (MPN), MDS/MPN, or otherwise unexplained cytopenia after receiving ^177^Lu-oxodotreotide [20]. The median latency period at diagnosis was 41 months. One case of CML was reported in a 61-year-old man with pancreatic NET who had developed it at 42.3 months after PRRT and had been receiving transarterial chemoembolization as a precursor treatment [20]. This case was identified as Ph+ (Philadelphia chromosome-positive). In another report, a 64-year-old patient with pancreatic NET, who had no prior chemotherapy but was treated with sunitinib, developed CML at 60 months after PRRT [22]. It has been reported that treatment with imatinib is successful. Furthermore, they reported that patients with PRRT-related MDS and AML had worse prognosis than those with de novo MDS and AML. Meanwhile, patients with CML had a similar prognosis, whether de novo or not, and tyrosine kinase inhibitor treatment was effective. These findings are like those of our study, in which the patient’s prognosis was not affected by the data cut-off period.

Notably, we did not observe any changes in renal function in this study. The main mechanism of renal damage is believed to be partly due to reabsorption and retention in the proximal tubular cells [23]. The results of this study support those of previous studies that reported that renal function deteriorated after exacerbation following PRRT. However, this deterioration was also due to the influence of other drug therapies and a decline in the patient’s general condition. Thus, the possibility of long-term prolongation of renal damage due to PRRT is extremely low [24].

The results of this study showed that PRRT is extremely useful and safe for long-term disease control in Japanese patients with NET. The use of PRRT as a first-line treatment has been established in some clinical trials. Nevertheless, it is necessary to conduct studies to confirm the long-term therapeutic efficacy and safety of first-line PRRT in Japan. Furthermore, this study provides important insights.

## Conclusions

PRRT with ^177^Lu-oxodotreotide demonstrated sustained antitumor efficacy and an acceptable long-term safety profile in Japanese patients with advanced, well-differentiated NETs. The median PFS period exceeded 3 years, and median OS period was not reached during follow-up, indicating durable disease control. Late adverse events were rare, with only one case of hematologic malignancy observed. These findings support PRRT as a key therapeutic option for somatostatin receptor–positive NETs in Japan and provide important evidence to guide clinical practice and future research on optimizing treatment strategies and safety monitoring.

## Supplementary Information

Below is the link to the electronic supplementary material.

Supplementary file1 (PDF 1138 kb)

## Data Availability

The data that support the findings of this study are not publicity available due to privacy reasons, but further enquiries can be directed to the corresponding author.
