# Supplementary material for: Long-term efficacy and safety of peptide receptor radionuclide therapy in Japanese patients with unresectable neuroendocrine tumor: extension of the Japanese phase I and phase I/II study
Source: Ann Nucl Med. 2026 Feb 12;40(6):687–94. doi: 10.1007/s12149-026-02169-1 (PMC13219169; doi:10.1007/s12149-026-02169-1)

Supplementary Table 1

Primary lesion	ORR , n (%)	DCR, n (%)	CR/PR/SD/PD, n (%)
Pancreas NET	8 (72.7)	10 (91)	1 (9.1)/7 (63.6)/2 (18.2)/1 (9.1)
Gastrointestinal NET	5 (62.5)	8 (100)	1 (12.5)/4 (50)/3 (37.5)/0 (0)
Gastric NET	1 (100)	1 (100)	0 (0)/1(100)/0 (0)/0 (0)
Small intestinal NET	2 (40)	5 (100)	1 (20)/1 (20)/3 (60)/0 (0)
Rectal NET	2 (100)	2 (100)	0 (0)/2 (100)/0 (0)/0 (0)
Lung NET	1 (50)	2 (100)	0 (0)/1 (50)/1 (50)/0 (0)

NET: Neuroendocrine tumor, ORR: Overall response rate, DCR: Disease control rate, CR: Complete response, PR: Partial response, SD: Stable disease, PD: Progressive disease,

Supplementary Figure 1

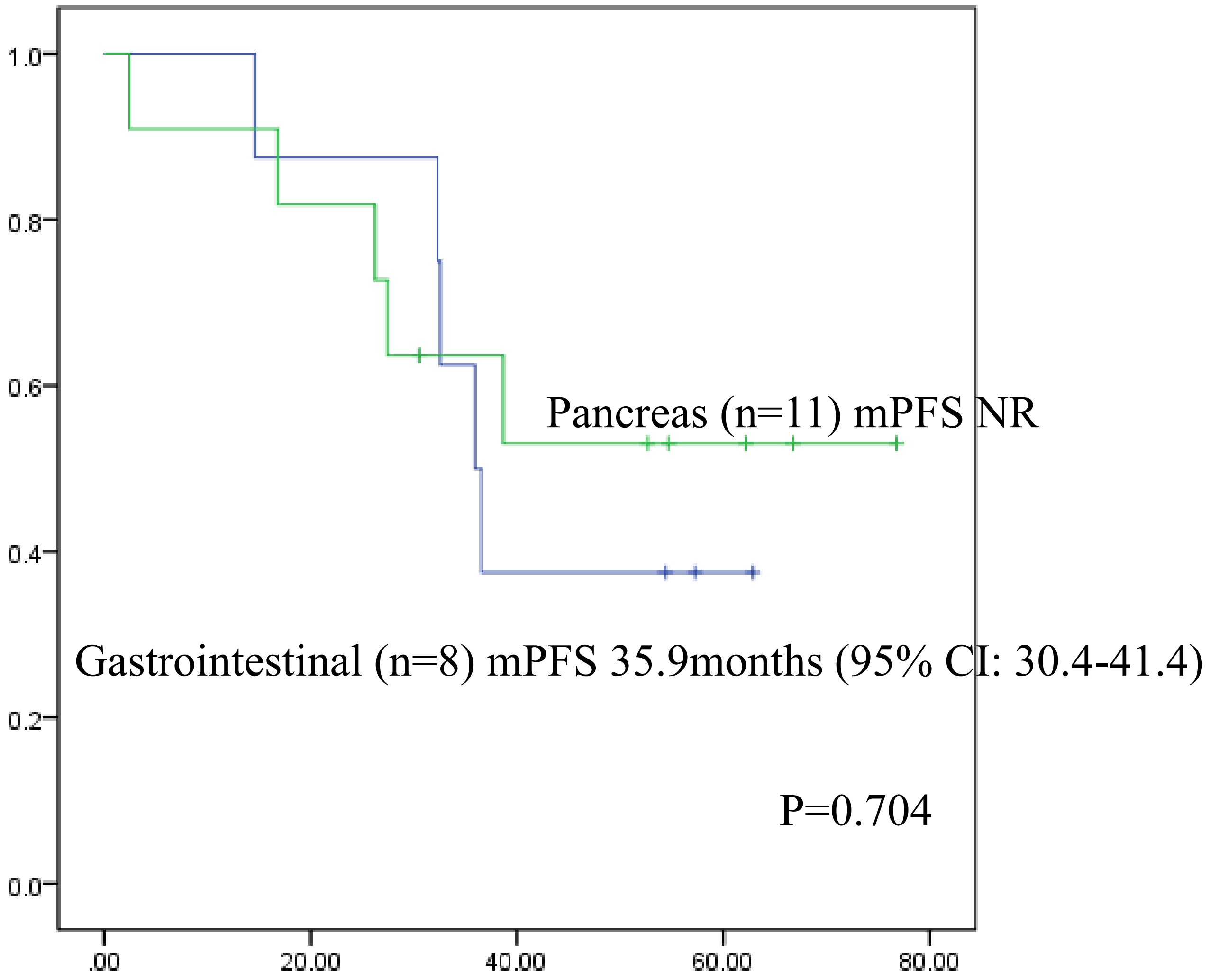

Supplementary Figure 2

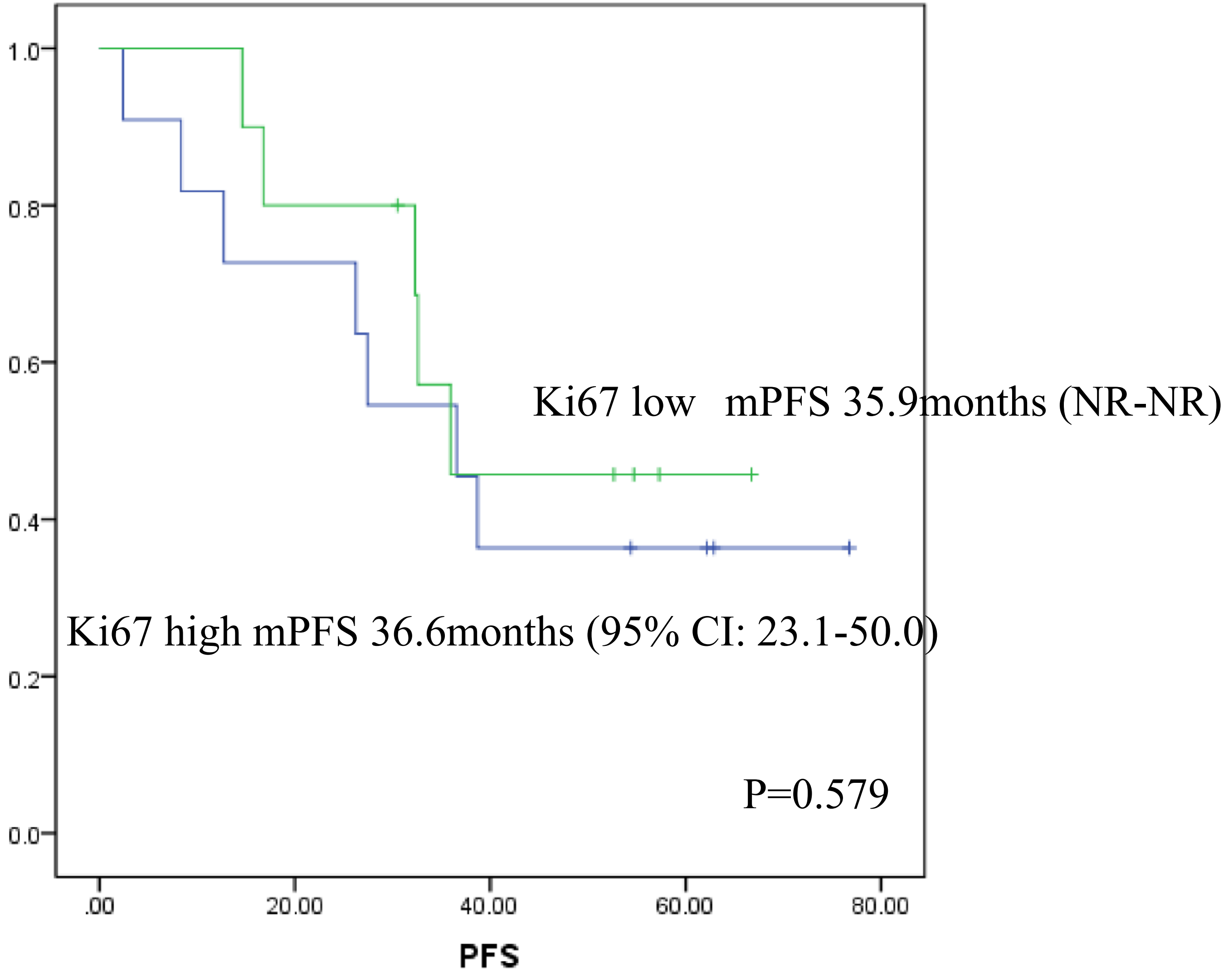

Supplementary Figure 3

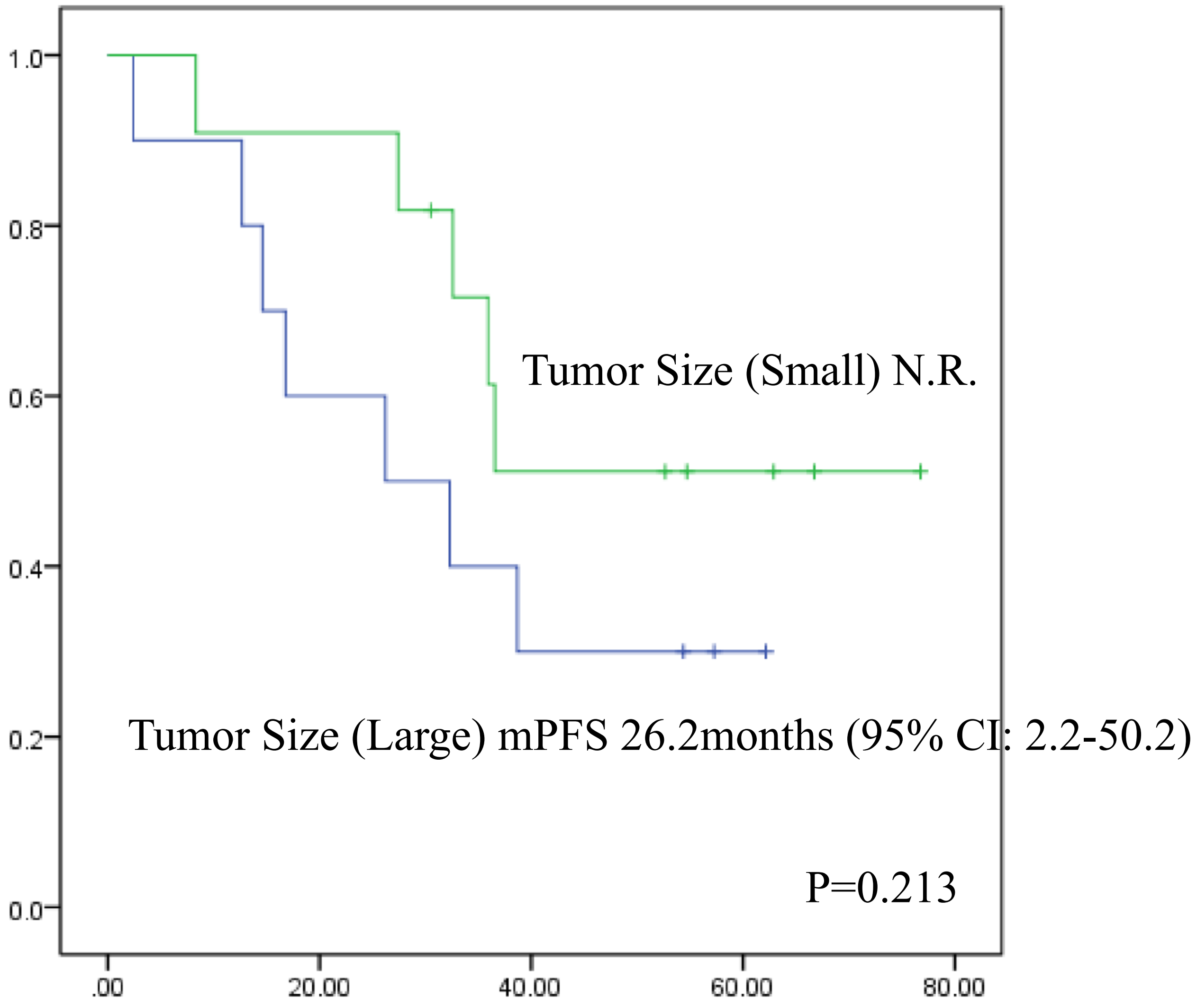

Supplementary Figure 4

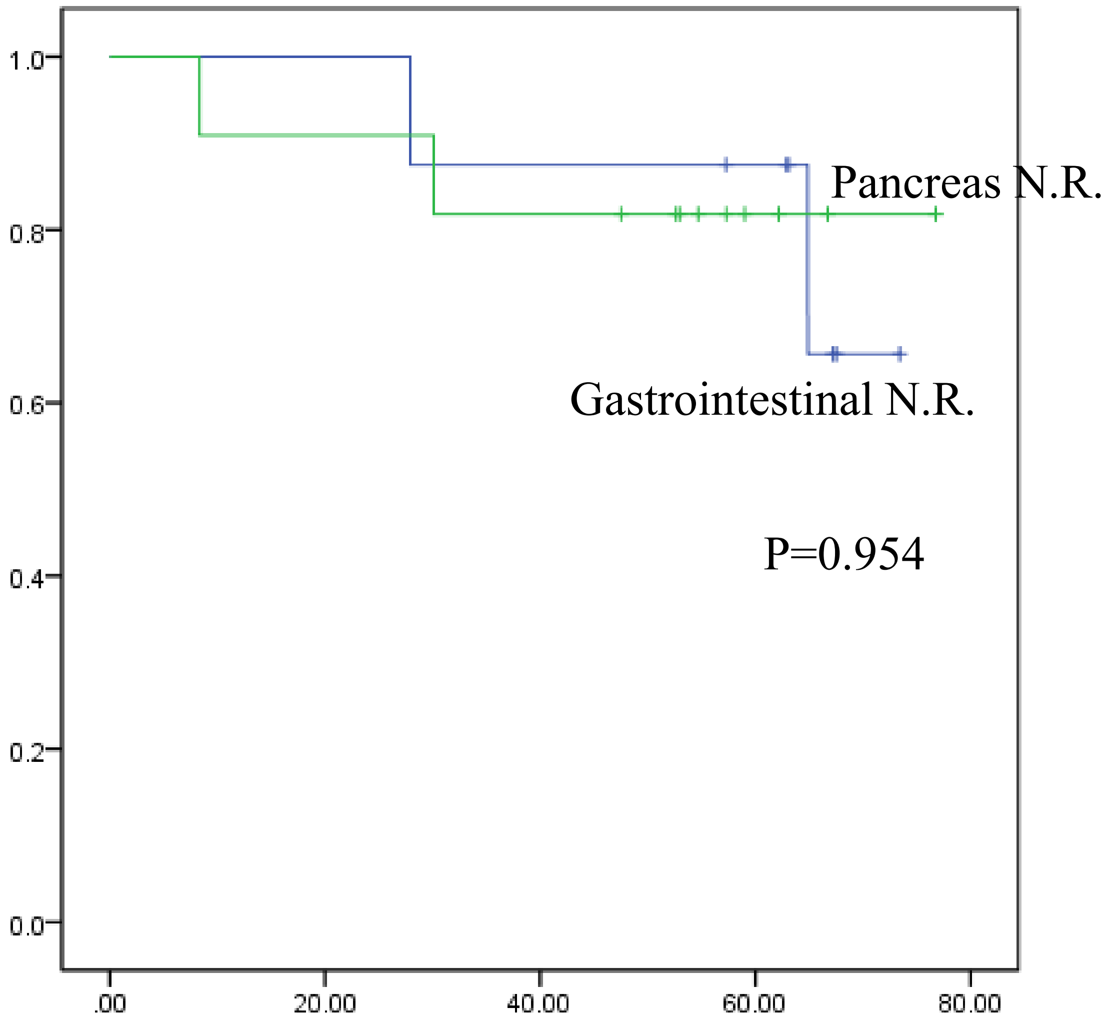

Supplementary Figure 5

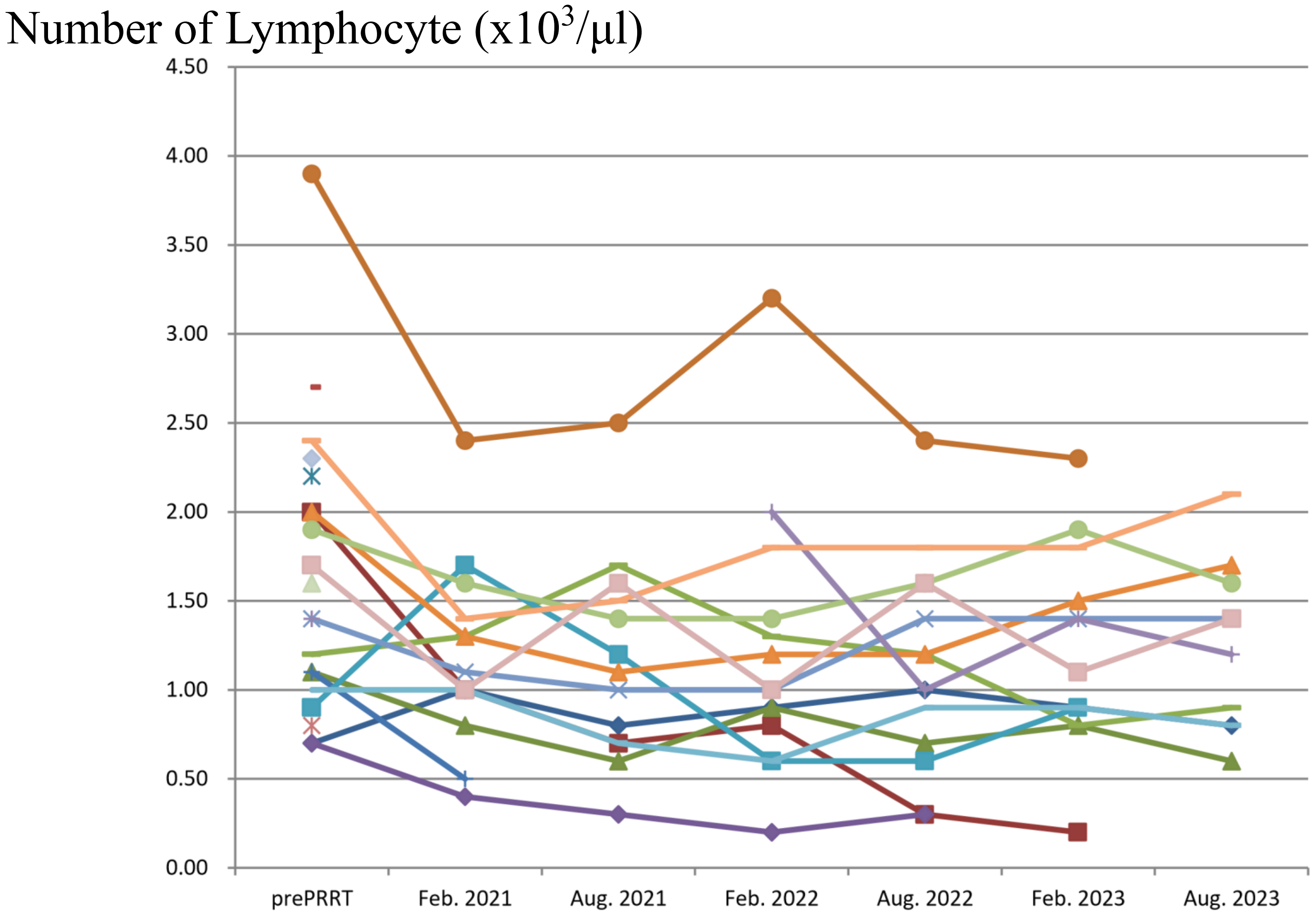

Supplementary Figure 6

Hemoglobin (g/dl)

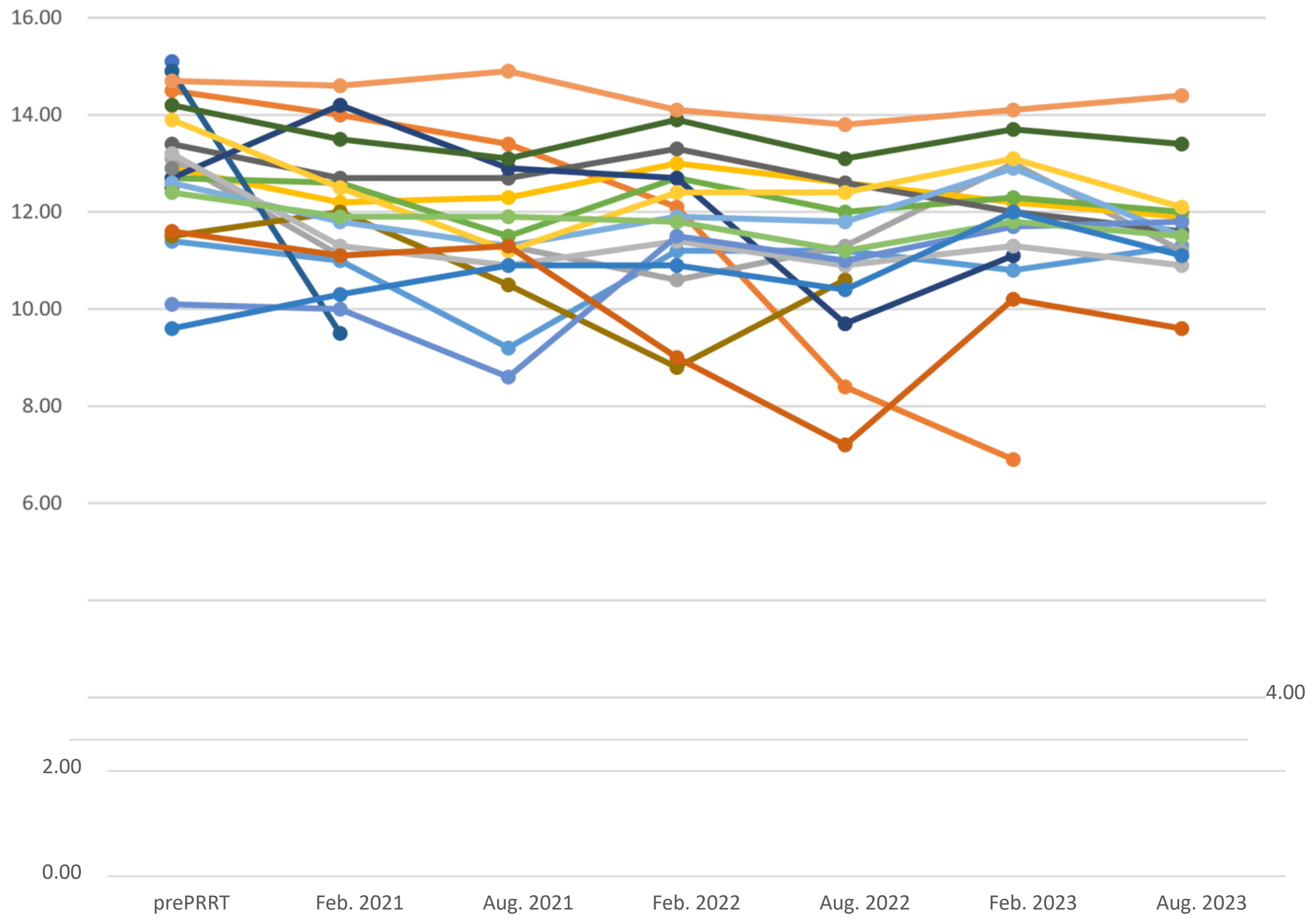

Supplementary Figure 7

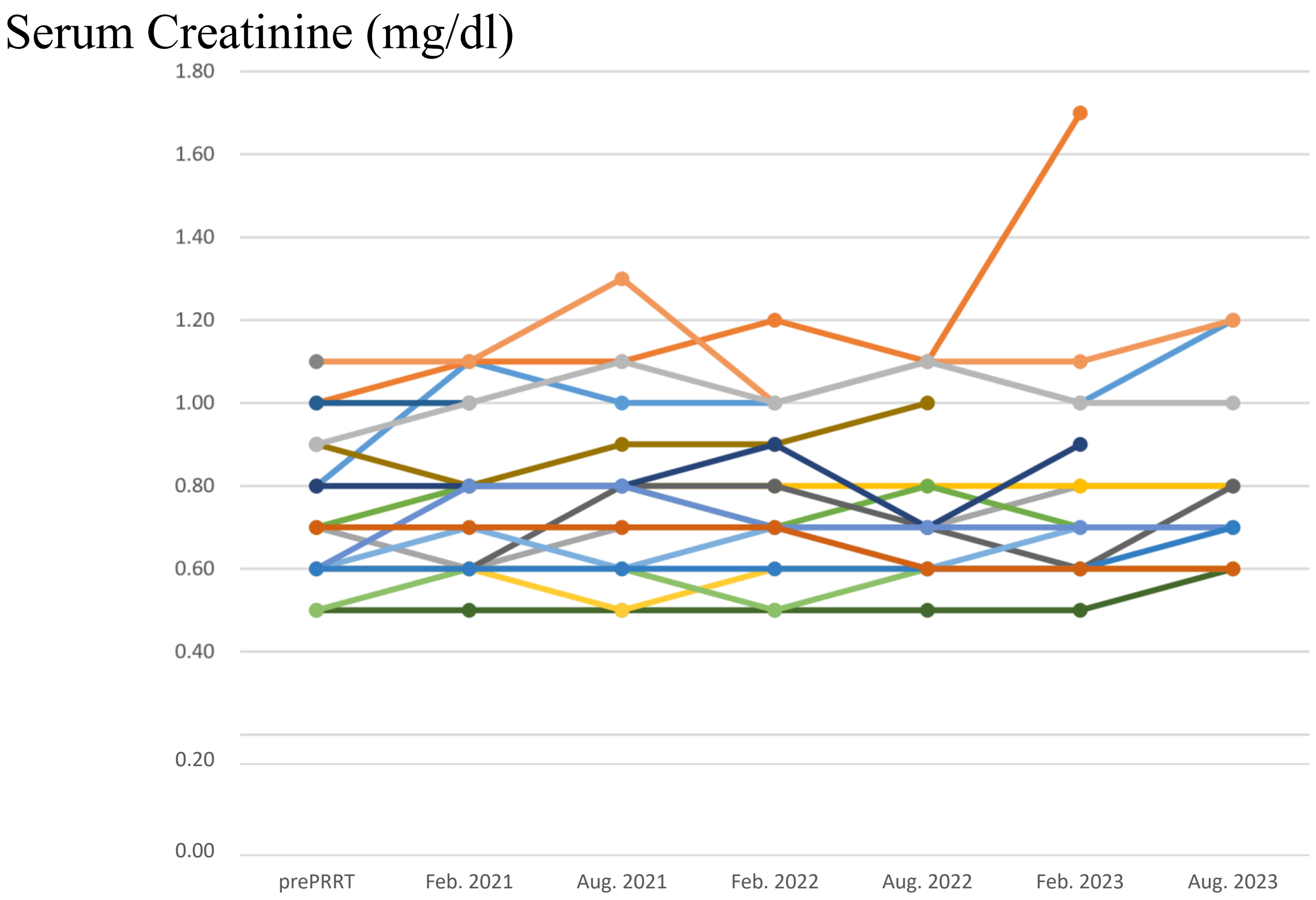

Supplementary Figure 8

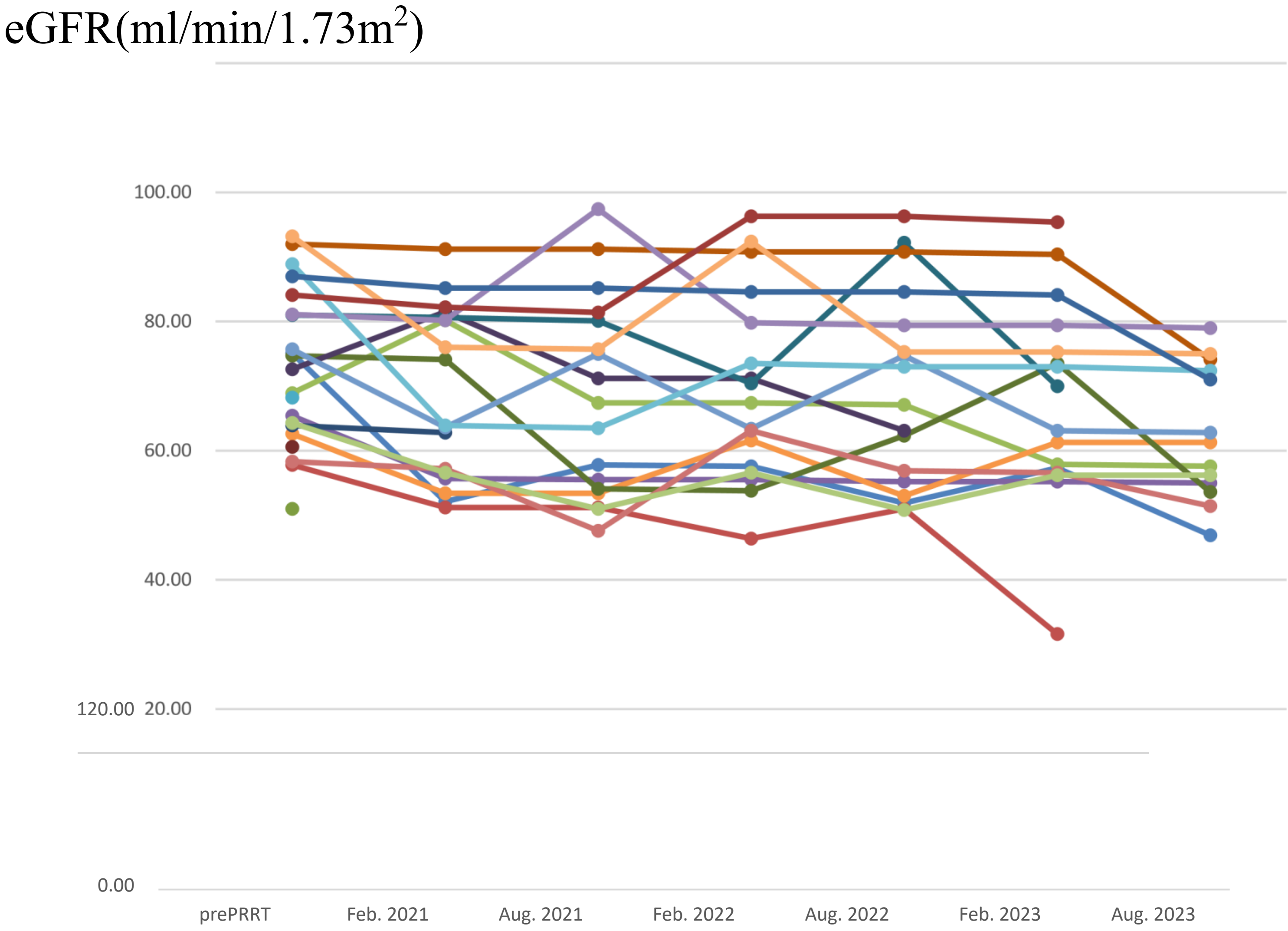

Supplement: Supplementary file 1 — Supplementary file1 (PDF 1138 kb) [file 12149_2026_2169_MOESM1_ESM.pdf]
